# Weaving the Net: The Interplay of Micro‐ and Macrosupport in ART Adherence

**DOI:** 10.1155/arat/5805050

**Published:** 2026-04-30

**Authors:** Kasim Abdulai, Ivan Addae-Mensah

**Affiliations:** ^1^ Department of Nutrition and Dietetics, Translational Nutrition Research Group, School of Allied Health Sciences, University of Cape Coast, Cape Coast, Ghana, ucc.edu.gh; ^2^ Hopespring Harvest Foundation, Cape Coast, Ghana; ^3^ Department of Population and Health, Faculty of Social Sciences, University of Cape Coast, Cape Coast, Ghana, ucc.edu.gh

**Keywords:** ART adherence, HIV, macrosupport, microsupport, social support

## Abstract

**Introduction:**

Sustained antiretroviral therapy (ART) adherence is fundamental to managing HIV, yet it remains a significant challenge, particularly in resource‐limited settings. Adherence is shaped by a complex interplay of support systems, which can be categorized as “microsupport”: immediate, informal assistance from personal networks, and “macrosupport”: formal, structural interventions from healthcare systems. The interaction and comparative impact of these two systems on long‐term adherence are not well understood.

**Objective:**

This study aimed to explore the lived experiences of people living with HIV in Ghana as they navigate both micro‐ and macrosupport systems to inform the development of more effective, multilevel adherence strategies.

**Methods:**

A qualitative descriptive design was employed at the Cape Coast Teaching Hospital in Ghana. Data were collected between June and September 2025 through semistructured in‐depth interviews and a focus group discussion with 21 purposively sampled adults who had been on ART for at least six months. Thematic analysis, facilitated by NVivo software, was used to analyze the data.

**Results:**

Analysis revealed that adherence is sustained by a delicate balance between the two support systems. Microsupport, manifesting as timely financial lifelines, spousal reminders, and quiet familial aid, was described as an immediate and critical “first line of defence” against missed doses. In contrast, macrosupport, such as peer groups and clinic‐based care, was valued for its broad reach but often perceived as unreliable due to structural inefficiencies, such as long wait times and inconsistent resource provision. Consequently, participants expressed a strong desire for combined approaches that integrate the personal, responsive nature of microsupport within the formal structure of macrosupport.

**Conclusion:**

Effective ART adherence depends not on choosing between micro‐ and macrosupport but on innovatively weaving them together. Microsupport provides an essential, agile safety net, while macrosupport offers a broader structural foundation. To build a resilient ecosystem of care, healthcare systems must formally recognize and bolster microsupport networks while making macrolevel interventions more reliable, personalized, and responsive to patient needs.

## 1. Introduction

The success of antiretroviral therapy (ART) in transforming HIV into a manageable chronic condition is entirely contingent upon sustained adherence, which remains a significant global challenge. While near‐perfect adherence (≥ 95%) is required for optimal viral suppression and preventing drug resistance, various barriers often impede this goal, particularly in resource‐limited settings [1–3]. The World Health Organization’s “treat‐all” approach has expanded access but has also heightened the need for robust support systems tailored to diverse populations who initiate treatment earlier and live longer with HIV [1, 4]. Understanding adherence requires a social–ecological perspective that recognizes the complex interplay between individual, interpersonal, community, and structural factors, including stigma, poverty, and gender inequality, which collectively influence a person’s ability to maintain treatment [1, 5, 6].

In Ghana, while the National AIDS Control Program has scaled up ART access, national adherence rates remain suboptimal, often fluctuating below the 95% threshold required for viral suppression [7]. Local macrosupport policies, such as the decentralization of care, have improved reach, yet their implementation is often hampered by systemic challenges including drug shortages and inconsistent resource provision [8]. Crucially, the interplay between these structural interventions and microlevel community assistance remains a critical gap in the HIV landscape, particularly in regions, such as the Central, where distinct rural–urban dynamics shape adherence outcomes [9].

At the interpersonal level, “micro‐support” refers to the immediate, practical assistance provided by an individual’s social network. This includes small, timely financial loans for transport or food, emotional encouragement, and daily reminders from family, partners, or friends [1, 10, 11]. Such support is highly adaptable and addresses critical logistical hurdles, such as the cost of traveling to a clinic or the need to take medication with food. Cohort studies have demonstrated that strong social support is a consistent predictor of better ART adherence and viral suppression, as it mitigates isolation and provides a direct safety net during times of crisis [1, 12, 13]. This “quiet” familial and community support often functions as a crucial first line of defense against missed doses.

In contrast, “macro‐support” encompasses structural interventions designed to create an enabling environment for adherence. These system‐level programs include clinic‐based adherence counseling, peer support groups, and the provision of material aid, such as food parcels or transport vouchers [1, 14]. While these initiatives have the potential to reach a broad population, their efficacy can be hampered by structural inefficiencies, such as long wait times, stock‐outs, and a lack of personalized care [14]. Evidence from randomized trials and systematic reviews on structural interventions shows mixed results, underscoring that their success depends heavily on reliable implementation and cultural relevance [15, 16]. The impersonal nature of some macrosupport can create a gap where patients’ specific, evolving needs are not fully met.

Given the respective strengths and limitations of micro‐ and macrosupport, a critical, underexplored question is how they interact and compare in their impact on long‐term adherence. Patients often express a desire for combined approaches that leverage the immediacy of microsupport with the broader resources of macrosupport [17]. However, when macrosupport systems are unreliable, individuals must rely more heavily on their personal networks, which may be insufficient during prolonged hardships [1, 18]. This qualitative inquiry aims to bridge this gap by directly exploring the lived experiences of individuals navigating both support systems, with the goal of informing more effective, multilevel strategies that ensure no patient falls through the cracks.

## 2. Methods

### 2.1. Study Design

This study employed a qualitative descriptive design to explore the complex, multifaceted nature of ART adherence among people living with HIV (PLWH) in Ghana. This approach was selected to capture rich, detailed insights into the lived experiences of participants, particularly focusing on the roles of “micro‐support” (informal, interpersonal support from family, friends, and social networks) and “macro‐support” (formal, structural support from clinics, programs, and institutions) in influencing adherence behaviors. The design utilized semistructured in‐depth interviews (IDIs) and focus group discussions (FGDs) to investigate key themes, including food insecurity, stigma, socioeconomic constraints, and health system interactions. This methodological choice allowed for flexibility in probing emergent themes and capturing nuanced realities, prioritizing depth of understanding over generalizable quantification [1].

### 2.2. Study Population

The study population comprised adults living with HIV (aged 18 years and older) who had been receiving ART for a minimum of 6 months and were actively receiving care at the Cape Coast Teaching Hospital (CCTH) in Ghana. This setting was chosen as it serves a diverse patient population from urban and peri‐urban areas, providing a representative sample of individuals facing varied socioeconomic challenges that influence ART adherence. Participants were required to be on ART for at least 6 months to ensure they had sufficient experience with treatment adherence challenges and support mechanisms.

### 2.3. Sample Size and Sampling Technique

A purposive sampling technique was used to recruit participants who could provide rich, information‐rich insights relevant to the study objectives. The final sample comprised 21 participants engaged in a combination of IDIs and one FGD. This number was not predetermined but was guided by the principle of thematic saturation, the point at which new data collection no longer yielded novel themes or insights but instead reinforced previously identified patterns. Thematic saturation was determined to be achieved after 20 individual interviews and one FGD (total *N* = 21). For sociodemographic characterization, the analysis reflects a total of 20 participants, as the sociodemographic data for one individual were incomplete. Purposive sampling ensured the inclusion of a diverse range of perspectives based on key characteristics, such as age, gender, and socioeconomic status to capture varied experiences related to ART adherence [1, 19].

### 2.4. Inclusion Criteria

Eligible participants were individuals aged 18 years or older who had been receiving ART for at least 6 months before the study. They were required to possess the capacity to provide informed consent, demonstrate effective communication skills in the study’s primary languages (English or Fante), and express willingness to participate in IDIs or FGDs.

### 2.5. Exclusion Criteria

Individuals were excluded from participation if they were under 18 years of age, lacked the capacity to provide informed consent due to significant cognitive impairment or acute psychiatric distress, or had been on ART for less than 6 months. Additional exclusion criteria included current hospitalization for a serious opportunistic infection or other acute medical condition, an inability to communicate in the study’s primary languages without an interpreter, and concurrent enrollment in another interventional study targeting ART adherence.

### 2.6. Data Collection Tools and Procedures

Primary data were collected through IDIs and FGDs. A semistructured interview guide was developed with open‐ended questions to explore participants’ perceptions of the connection between nutrition and ART, daily routines, social supports, and contextual barriers and facilitators associated with treatment adherence. The guide was developed based on the study objectives and a review of existing literature on ART adherence in low‐ and middle‐income countries (LMICs) [1]. It was piloted before data collection to ensure clarity and relevance.

All interviews and discussions were conducted in a private setting at the CCTH to ensure confidentiality and comfort. Sessions were audio‐recorded with participants’ permission and supplemented with field notes to capture nonverbal cues and contextual details. Data collection continued iteratively until thematic saturation was confirmed. On average, IDIs lasted 45–60 min, while the FGD lasted approximately 90 min. Participants were compensated for their time with a small stipend to cover transportation costs, a practice consistent with ethical research conduct in resource‐limited settings [19, 20].

### 2.7. Reflexivity and Researcher Positionality

The research team consisted of faculty and researchers from the University of Cape Coast, specializing in Public Health Nutrition and Dietetics. As established academics within the Ghanaian healthcare landscape, the researchers maintained a reflexive stance to mitigate the interviewer effect, where participants might provide socially desirable answers. To ensure rigor, interviews were conducted in private settings by researchers not directly involved in the participants’ clinical care, emphasizing that participation would not affect their treatment.

### 2.8. Data Analysis

All audio‐recordings were transcribed verbatim to ensure accuracy. Transcripts were translated into English where necessary by a bilingual researcher to maintain consistency in analysis. Thematic analysis, following the six‐phase framework by Braun and Clarke [21], was employed as the primary analytical method [21]. The process was facilitated using NVivo software (Version 12) to manage and organize the qualitative data.

The analysis began with repeated reading of transcripts to ensure familiarity with the data. Initial codes were generated inductively from the data, focusing on participants’ descriptions of microsupport (e.g., financial lifelines and emotional support) and macrosupport (e.g., structural programs and clinic‐level support). These codes were then collated into potential themes, which were reviewed, refined, and defined through a collaborative process between researchers to ensure consistency and credibility. The final step involved weaving the themes into a coherent narrative, supported by direct excerpts from the participants [1, 19].

### 2.9. Ethical Considerations

Ethical approval for this study was obtained from the CCTH Institutional Review Board (CCTHERC/EC/2025/184). The research was conducted in full accordance with established ethical principles and the requirements of this review board. All participants provided written informed consent after receiving a comprehensive explanation of the study’s purpose, procedures, potential risks, and benefits. The autonomy of participants was rigorously respected through the emphasis that participation was entirely voluntary and that they could withdraw at any time without any penalty or effect on their medical care.

Confidentiality was protected through the assignment of anonymized ID codes, secure password‐protected storage of all electronic data and physical transcripts, and the reporting of only aggregate, de‐identified findings in any research outputs. To minimize potential harm, interviewers were trained to handle sensitive topics with empathy and discretion, and procedures were established to provide immediate referrals to counseling or support services should any participant experience significant distress. The conduct of the study was continuously monitored to ensure that the rights, dignity, and well‐being of all participants remained the paramount consideration throughout the entire research process [20, 22].

## 3. Results

### 3.1. Sociodemographic Characteristics of the Study Sample

As shown in Table 1, the study participants (*N* = 20) were predominantly female (85%), with a mean age of 41.9 years (SD = 12.56). Our sample was predominantly female, which may influence the transferability of findings, particularly around themes of childcare and gender‐specific economic roles. Future research should actively recruit more male participants. The sample exhibited significant economic diversity, with a median monthly household income of 1200.0 Ghana cedis (IQR = 600.0–1850.0). At the time of the study, 1 USD was equivalent to an average of 11.205 GHS. Education levels were generally low, with 70% having completed junior high school or less. Reflecting this, common occupations were trading (40%) and farming or unemployment (15% each). Most lived in compound houses (70%) and relied primarily on purchased sachet or bottled water (80%) for drinking, while sanitation facilities were split between flush toilets (45%) and various types of latrines (50%) [1].

**TABLE 1:** Sociodemographic characteristics of the study sample (*N* = 20).

Characteristic	Frequency (*n*)	Percentage (%)
Gender		
Female	17	85.0
Male	3	15.0
Marital status		
Married	8	40.0
Single	6	30.0
Divorced	4	20.0
Widowed	2	10.0
Cohabiting	1	5.0
Highest education		
Primary	6	30.0
Junior high school (JHS)	5	25.0
Senior high school (SHS)	3	15.0
Tertiary (Diploma/BSc)	3	15.0
Higher^a^	2	10.0
No formal schooling	1	5.0
Occupation		
Trader	8	40.0
Unemployed	3	15.0
Civil servant	3	15.0
Farmer	3	15.0
Self‐employed	1	5.0
Food vendor	1	5.0
Laborer	1	5.0
Monthly household income (GHS)		
Median (IQR)	1200.0 (600.0–1850.0)	
Range	0.0–4500.0	
Primary income source		
Business	12	57.14
Remittances	5	23.81
Other	4	19.05
Farming	3	14.29
Salary/wages	2	9.52
Housing type		
Compound house	14	70.0
Flat/apartment	4	20.0
Self‐contained	2	10.0
Main water source		
Sachet/bottled	16	80.0
Pipe‐borne	2	10.0
Borehole	1	5.0
Other^b^	1	5.0
Sanitation facility		
Flush toilet	9	45.0
Pit latrine	4	20.0
VIP latrine	6	30.0
Others^c^	1	5.0

Abbreviations: GHS = Ghana Cedis, SD = standard deviation.

^a^“Higher” education was specified by participants and is distinct from “Tertiary” in this dataset.

^b^One participant’s response for water source was blank in the provided data.

^c^One participant’s response for the sanitation facility was blank in the provided data. Percentages may not sum to 100 due to rounding.

Analysis of the interviews revealed that ART adherence is profoundly influenced by two interconnected support systems: microsupport (informal, interpersonal support) and macrosupport (formal, structural support). Participants described how the immediacy and flexibility of microsupport often addressed critical, day‐to‐day barriers, while macrosupport provided essential, though sometimes inconsistent, structural safety nets. The comparative efficacy of these supports and participant preferences for a combined approach emerged as central themes. Key findings are summarized in Table 2, which presents the thematic structure along with illustrative participant excerpts.

**TABLE 2:** Themes and illustrative quotes on microsupport and macrosupport in ART adherence.

Theme	Subtheme	Identified codes	Participant excerpt
Microsupport	Financial lifelines	Small, immediate monetary help	13,003: “ I called my friend, and he showed up 5 minutes later with GHS 100 tucked in his pocket (approximately USD 8.19). He said, ‘Go get your pills; worry about paying me back after your next sale’.”
			32,015: “I have one of his friend’s numbers… I called and they sent me 1000.00 (approximately USD 81.93) to use for buying and selling.”
			32,002: “If the market is slow, I will sell more cassava earlier in the month or borrow a little from a neighbor and pay back after market.”
	Emotional and practical support	Spousal reminders and nurturing	13,003: “My wife reminds me each morning even before I finish praying.”
			33,002: “My wife supports me the most. She reminds me and encourages me.”
			13,004: “My husband is my rock. He reminds me to take my medication and always checks in.”
	Quiet familial support	Unspoken but critical help	13,003: “My mother, God bless her, quietly ensures there’s always a little something on the table when possible.”
			32,002: “My son calls sometimes to ask if I have taken the medicine… He sends a little money if I tell him the transport is tight.”
			33,001: “My children make sure there’s food in the house for me to always be able to take my drugs.”

Macrosupport	Structural programs	Peer support groups	13,003: “I attend monthly peer‐led meetings at the clinic. They share experiences, tips for side effects, and sometimes distribute fortified porridge mix or small food parcels.”
			14,001: “Yeah, I’ve been to a few support groups. They’re really helpful. We share our experiences and support each other.”
			13,003: “At one meeting, they gave out 3 kg of soy‐enriched maize flour that fed my family for 2 weeks.”
	Clinic‐ and system‐level support	Personalized, nonjudgmental care	13,003: “the adherence counselor calls me if I miss an appointment… She’ll ask, ‘Is everything okay?’ and listen without judgment.”
			32,011: “They consistently call to check up, support and encourage me.”
			13,001: “The clinic staff have been really great. They’re always encouraging me and supporting me, especially when I had a rough patch and stopped taking my meds.”
	Material and resource support	Food parcels and transport vouchers	13,003: “subsidized transport vouchers tied to clinic visits, a weekly small food package of staples like maize and beans, and private counseling rooms at the clinic.”
			32,002: “Help with transport, even a small voucher or monthly support, would change things. Also, more small talks about cheap nutritious foods.”
			13,004: “Pooled transport with my neighbor and adjust meals using pantry staples like beans. Those small adaptations keep me consistent.”

Comparative efficacy	Immediate vs. sustained impact	Microsupport: immediate but variable	13,003: “That simple act [friend lending GHS 100] kept me from missing a refill and reminded me I’m not alone.”
			32,002: “I would rather skip some small things at home than stop the medication.”
			13,006: “I never sacrifice my medicine refills.”
		Macrosupport: broader but less personalized	13,003: “Peer‐led meetings… share experiences and tips, but sometimes the food parcels run out.”
			12,001: “No stock‐outs, no paperwork delays. They even send me a reminder SMS 3 days or even call before my refill date.”
			32,013: “I think when they are giving the date, they have to cross check that, that is the correct date. Because you will come here and then there is nobody you have to go back. ”
	Barriers to macrosupport	Structural inefficiencies	13,003: “the wait times are consistently long sometimes up to 3 hours in the queue under the hot sun. The building has no proper waiting shade or enough chairs.”
			32,012: “They delay too much, when I come, they talk a lot instead of attending to us.”
			32,013: “There was once when they were on strike so they asked me to go to the hospital at Baakano for the medicine.”
	Participant preferences	Desire for combined approaches	13,003: “A mentor who’s been through this could teach practical ways to deal with stigma, share tips for cheap nutrient‐dense foods, and offer emotional support. I’d attend every session if mentors were available every week.”
			13,002: “I think it would be helpful to have someone who understands what I’m going through.”
			13,007: “Peer mentors could remind people about medication, give advice, and support adherence. That would help a lot.”

### 3.2. The Critical Role of Microsupport

Microsupport, characterized by its immediacy and personal nature, was frequently described as the first line of defense against missed doses. This support manifested in three primary ways.

Financial lifelines from personal networks were crucial in overcoming acute financial shocks. This involved small, timely loans or gifts that directly enabled clinic visits or medication refills, as one participant noted: “*I called my friend, and he showed up five minutes later with GHS 100 tucked in his pocket. He said,* ‘*Go get your pills; worry about paying me back after your next sale*’*.* ” (13,003).

Beyond financial aid, emotional and practical support from spouses was a cornerstone of daily adherence. This often took the form of consistent reminders and nurturing, which reduced the mental burden on participants. A participant shared, “*My wife reminds me each morning even before I finish praying*” (13,003), highlighting how this support was seamlessly integrated into daily routines.

Finally, quiet familial support was a vital, often unspoken, form of assistance. Family members, including children, provided critical help with food and transportation, directly addressing key barriers to adherence. One participant explained, “*My children make sure there’s food in the house for me to always be able to take my drugs*” (33,001).

### 3.3. The Broad Yet Fragile Nature of Macrosupport

In contrast, macrosupport from institutional programs was valued for its broader reach but was often perceived as less consistent and personalized.

Structural programs, such as peer support groups, were appreciated for both the material resources and shared experiences they provided. As one participant stated, “*Yeah, I’ve been to a few support groups. They’re really helpful. We share our experiences and support each other*” (14,001). The distribution of food parcels at these meetings was a particularly valued benefit.

The quality of clinic‐ and system‐level support was a significant factor. Participants expressed deep appreciation for healthcare providers who offered personalized, nonjudgmental care, especially during difficult times. This was encapsulated by one participant’s experience: “*The clinic staff have been really great. They’re always encouraging me and supporting me, especially when I had a rough patch and stopped taking my meds*” (13,001).

However, the provision of material and resource support was inconsistent. While the idea of programs, such as transport vouchers and food packages, was highly desired, their inconsistent availability limited their impact.

### 3.4. Comparative Efficacy and Participant Preferences

A clear tension emerged between the two support systems. Participants characterized microsupport as immediate but variable, relying on the strength and capacity of one’s social network. Conversely, macrosupport was seen as broader but often hampered by structural inefficiencies. Long wait times and logistical problems were major barriers, with one participant citing “*wait times… up to 3 hours in the queue under the hot sun*” (13,003) as a significant deterrent to clinic attendance.

Ultimately, participants did not see these supports as mutually exclusive but complementary. There was a strong desire for combined approaches that leverage the strengths of both. Many participants proposed a model that integrates the personal touch of microsupport within the macrosupport system, such as through peer mentorship programs. One participant envisioned, “*A mentor who’s been through this could teach practical ways to deal with stigma, share tips for cheap nutrient-dense foods, and offer emotional support. I’d attend every session if mentors were available every week*” (13,003). This synthesis was identified as the most promising path toward improving ART adherence.

## 4. Discussion

This study paints a clear picture: staying on HIV treatment is not just about individual willpower. It is about navigating a whole ecosystem of support, which our participants described as two main types. On one hand, there is the immediate, personal help from family and friends (microsupport). On the other hand, there is the broader, structural help from clinics and programs (macrosupport). Our findings show that both are essential, but their success hinges on how well they work together and how reliable they are in a person’s daily life.

First, the personal touch makes a huge difference. The small, timely loans from a friend for taxi fare or the daily reminders from a spouse were often what stood between our participants and a missed dose. This is not just a nice‐to‐have; research in Ghana confirms it. One study found that having someone to support your treatment makes you nearly three times more likely to stay on track, and those who get reminders from their network are almost twice as likely to adhere [23, 24]. However, this personal safety net has a weakness; it can fray under pressure. If a family faces a long stretch of unemployment or a bad harvest, that well of support can run dry, leaving individuals dangerously exposed [24].

At the same time, the larger support systems meant to provide a safety net often have holes in them. Our participants talked about clinic programs that were helpful in theory, such as peer groups that offered food parcels or transport vouchers, but were let down by reality. Long waits, stock‐outs, and other logistical headaches made this “macro‐support” feel unreliable. This is a common problem in many regions, where the best‐designed programs can fail if the health system itself is struggling with basic inefficiencies [25, 26]. For macrosupport to truly work, it needs to be consistently available and tailored to the community it serves [1].

To understand why our participants relied so heavily on microsupport, you have to look at their life circumstances. Most were women with little formal education and unstable incomes. For them, poverty is not a background factor; it is the main obstacle. A sudden need for 20 cedis for transport can be a crisis that threatens their treatment. Studies back this up, consistently linking low income to difficulties with adherence [27]. The fact that our group was mostly women also shapes the story, as gender roles often mean women bear the brunt of caregiving and economic stress, which influences how they both give and receive support [1, 28].

The most powerful insight from our participants was their desire for a combined approach. They did not want to choose between a caring friend and a well‐run clinic; they wanted both. They envisioned a system where a peer mentor, someone who’s been through it themselves, could offer both a listening ear and practical tips for getting cheap, nutritious food. This idea of blending the personal with the structural is supported by research that calls for multilevel solutions [1]. Programs that focus only on big‐picture policy often miss the daily struggles that wear people down, while those that only offer personal support cannot fix broken systems [29].

It is crucial to distinguish between the failure of support programs and the failure of general health infrastructure. For instance, participants who possessed robust microsupport, such as daily spousal reminders, still experienced adherence lapses when faced with macrolevel systemic failures, such as drug stock‐outs or clinical strikes. This finding aligns with evidence from southern Africa, where public sector strikes disrupted ART delivery and left patients unable to access treatment despite their personal commitment to adherence [30]. Similarly, research in resource‐poor settings has documented that supply problems and inconsistent drug availability create vulnerabilities that undermine treatment effectiveness, even among highly motivated patients [31]. This suggests that while weaving micro‐ and macrosupport is vital, neither can compensate for a lack of basic pharmaceutical security.

Our findings call for a smart redesign of HIV care support. Policy should officially recognize the power of microsupport by funding trained peer mentors who can offer that immediate, personal connection. At the same time, clinics and governments must fix the basic failures, such as drug stock‐outs and endless queues, that make macrosupport untrustworthy. A simple but powerful step would be to provide flexible, small cash vouchers at the clinic, acting with the speed of a friend’s loan but with the backing of the system. The goal is clear: to weave together the strength of the community with the resources of the system, creating a safety net that is both strong and responsive, so that no one has to manage their HIV treatment alone.

## 5. Conclusion

In conclusion, this study demonstrates that ART adherence is sustained through a delicate balance of informal microsupport and formal macrosupport. Microsupport provides the essential agile, daily safety net, while macrosupport offers a broader, though often fragile, structural foundation. The most profound insight from our participants is that the future of effective adherence support does not lie in choosing one over the other, but in innovatively weaving them together. By building macrosystems that are more personalized and responsive, and by formally recognizing and bolstering microsupport networks, healthcare systems can create a more resilient and effective ecosystem of care for people living with HIV.

### 5.1. Recommendations for Future Research

Based on the findings of this qualitative inquiry, several targeted avenues for future research are recommended to build upon and extend our understanding of support systems for ART adherence. First, a dedicated gender‐specific inquiry is essential to actively recruit male participants and explore potential divergences in how men experience and utilize both micro‐ and macrosupport, thereby ensuring that future interventions are equitable and effective across genders. Second, the participant‐proposed hybrid model of peer mentorship, which integrates the immediacy of microsupport with the structure of macrosupport, should be formally developed and its efficacy rigorously evaluated through randomized controlled trials (RCTs) measuring objective adherence metrics and viral suppression rates. Furthermore, employing longitudinal qualitative designs would illuminate the dynamic nature of support, capturing how individuals’ reliance on these different networks fluctuates over time in response to changing life circumstances, economic shocks, and health status. Collectively, these research directions would move beyond description toward the generation of robust evidence, ultimately guiding the development of more nuanced, adaptable, and effective support strategies that are deeply informed by the lived realities of people living with HIV in similar socioeconomic contexts.

### 5.2. Limitations of the Study

This study’s limitations should be acknowledged. The predominantly female sample (85%) may limit the transferability of findings to men, whose experiences with HIV support could differ. Furthermore, being conducted at a single tertiary hospital in Cape Coast affects the geographical generalizability of results to other regions or countries with different health systems and cultures. As a qualitative study, social desirability bias may also be present, where participants over‐reported adherence or gave provider‐favorable responses. Thus, while providing deep insights, these findings are not universally representative but offer a foundational perspective for future research across more diverse settings.

## Author Contributions

Conceptualization: Ivan Addae‐Mensah and Kasim Abdulai. Methodology: Ivan Addae‐Mensah and Kasim Abdulai. Investigation: Kasim Abdulai. Formal Analysis: Kasim Abdulai and Ivan Addae‐Mensah. Data Curation: Kasim Abdulai. Writing–original draft: Ivan Addae‐Mensah and Kasim Abdulai. Writing–review and editing: Ivan Addae‐Mensah and Kasim Abdulai. Supervision: Kasim Abdulai. Project administration: Ivan Addae‐Mensah and Kasim Abdulai.

## Funding

No funding was received for this manuscript.

## Disclosure

All authors have agreed to be accountable for the research presented. The affiliation of the corresponding author with HopeSpring Harvest Foundation is declared for transparency; however, the Foundation had no role in the design of the study; in the collection, analyses, or interpretation of data; in the writing of the manuscript; or in the decision to publish the results. The research was conducted, and the manuscript was prepared entirely independently, and the views expressed are solely those of the authors. This statement is made in accordance with the journal’s publication ethics policy.

## Conflicts of Interest

The authors declare no conflicts of interest.

## Data Availability

The qualitative data (anonymized transcripts) generated during this study are not publicly available due to the sensitive nature of HIV‐related research and the terms of the informed consent. However, they are available from the corresponding author on reasonable request for the purpose of secondary analysis or replication.
